# Supplementary material for: Structural basis for the promiscuous PAM recognition by Corynebacterium diphtheriae Cas9
Source: Nat Commun. 2019 Apr 29;10:1968. doi: 10.1038/s41467-019-09741-6 (PMC6488586; doi:10.1038/s41467-019-09741-6)
Supplement: Supplementary file 1 — Supplementary Information [file 41467_2019_9741_MOESM1_ESM.pdf]

Structural basis for the promiscuous PAM recognition by *Corynebacterium diphtheriae* Cas9

S. Hirano et al.

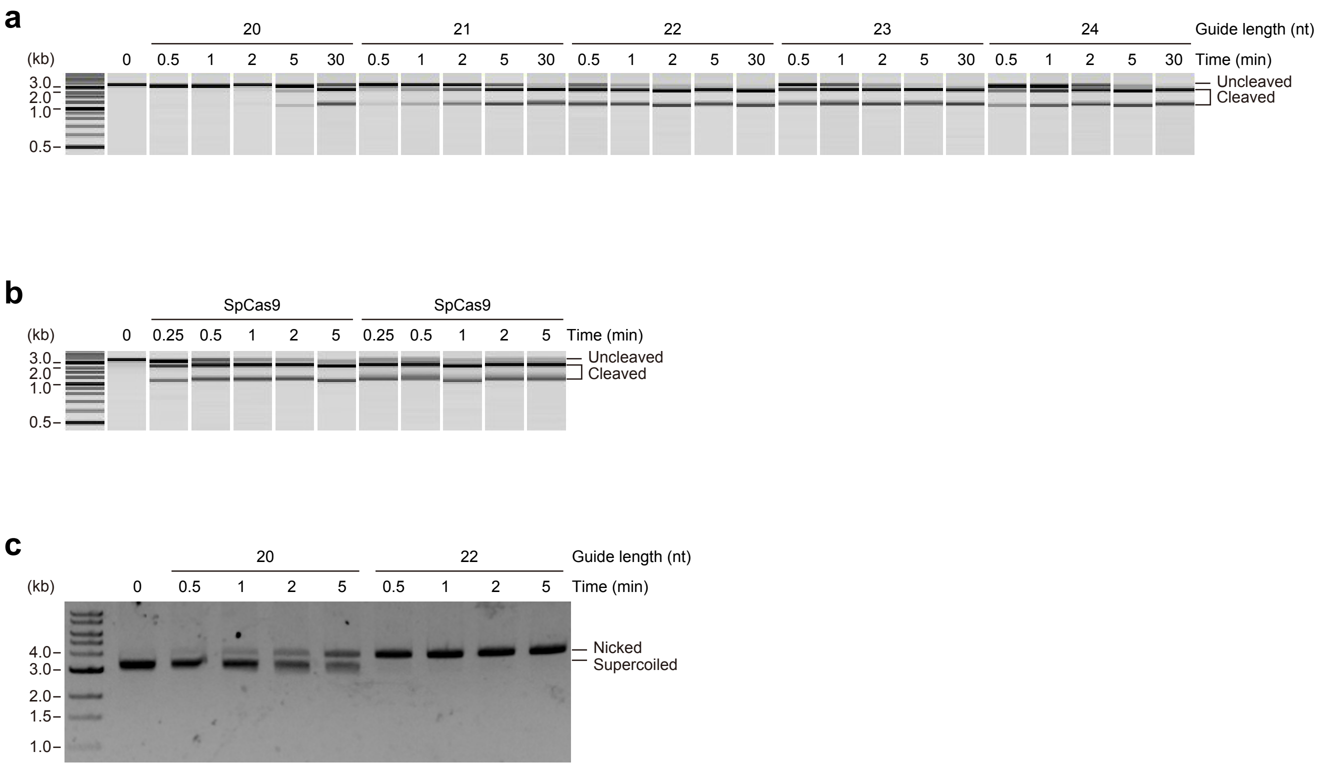

Supplementary Figure 1 | *In vitro* DNA cleavage activities of CdCas9.

a DNA cleavage time courses of CdCas9 with the 20–24-nt guide sgRNAs toward the linearized plasmid target with the GGGAAAC PAM (0.5, 1, 2, 5, and 30 min).

b DNA cleavage time courses of CdCas9 and SpCas9 toward the linearized plasmid target with the GGGAAAC PAM (0.25, 0.5, 1, 2, and 5 min). In (**a**) and (**b**), the reaction products were resolved, visualized, and quantified with a MultiNA microchip electrophoresis device.

c DNA cleavage time courses of the CdCas9 D10A mutant with the sgRNA20 or sgRNA22 toward the circular plasmid target with the GGGAAAC PAM (0.5, 1, 2, and 5 min). The reaction products were resolved on an ethidium bromide-stained 1% agarose gel, and then visualized using an Amersham Imager 600. Source data are provided as a Source Data file.

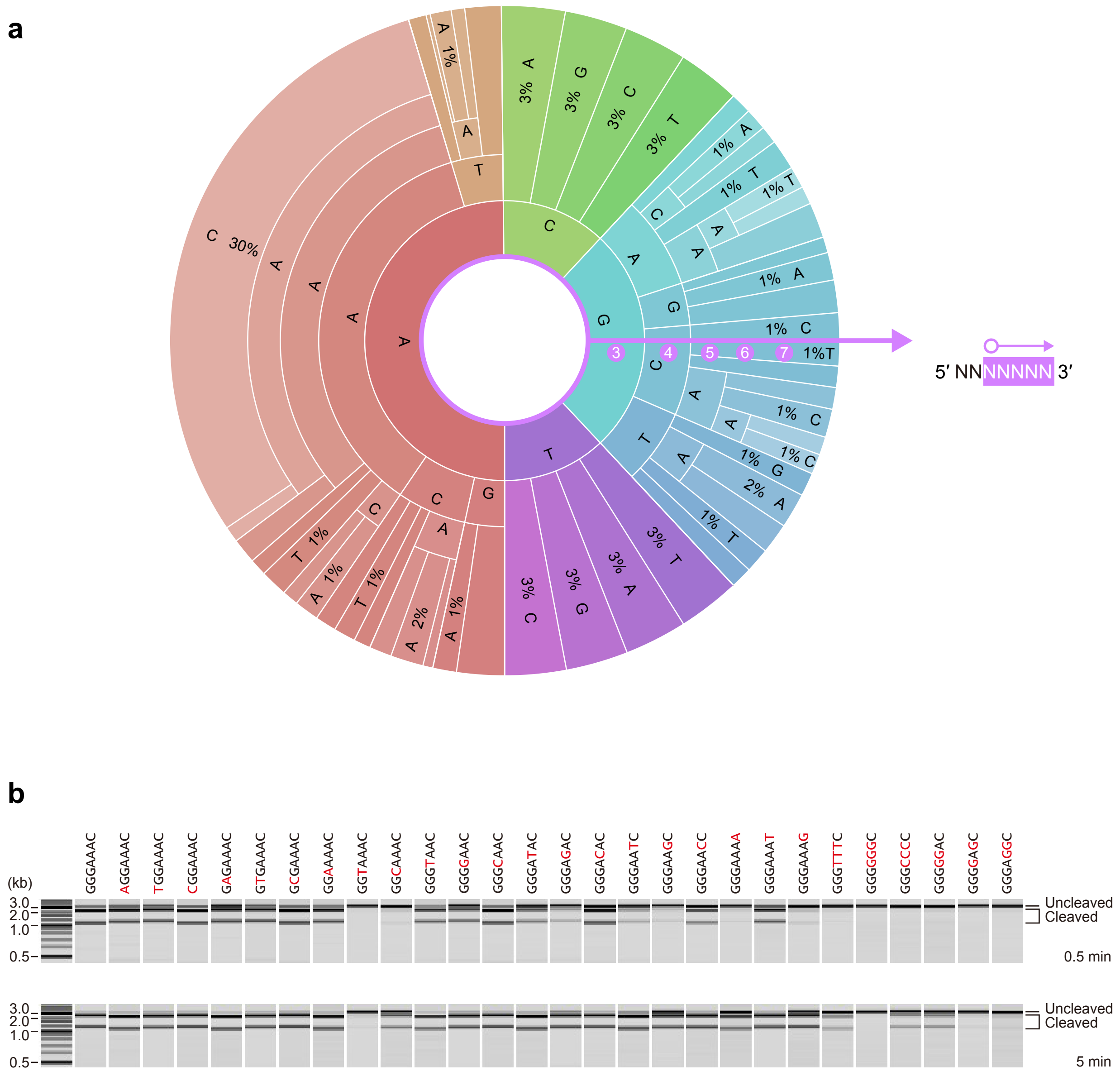

Supplementary Figure 2 | PAM preference of CdCas9.

a PAM wheel showing the results of the PAM identification assay. The area of the sector indicates the relative enrichment in the library.

b DNA cleavage time courses of CdCas9-sgRNA22 toward the linearized plasmid targets with the different PAMs (0.5 and 5 min).

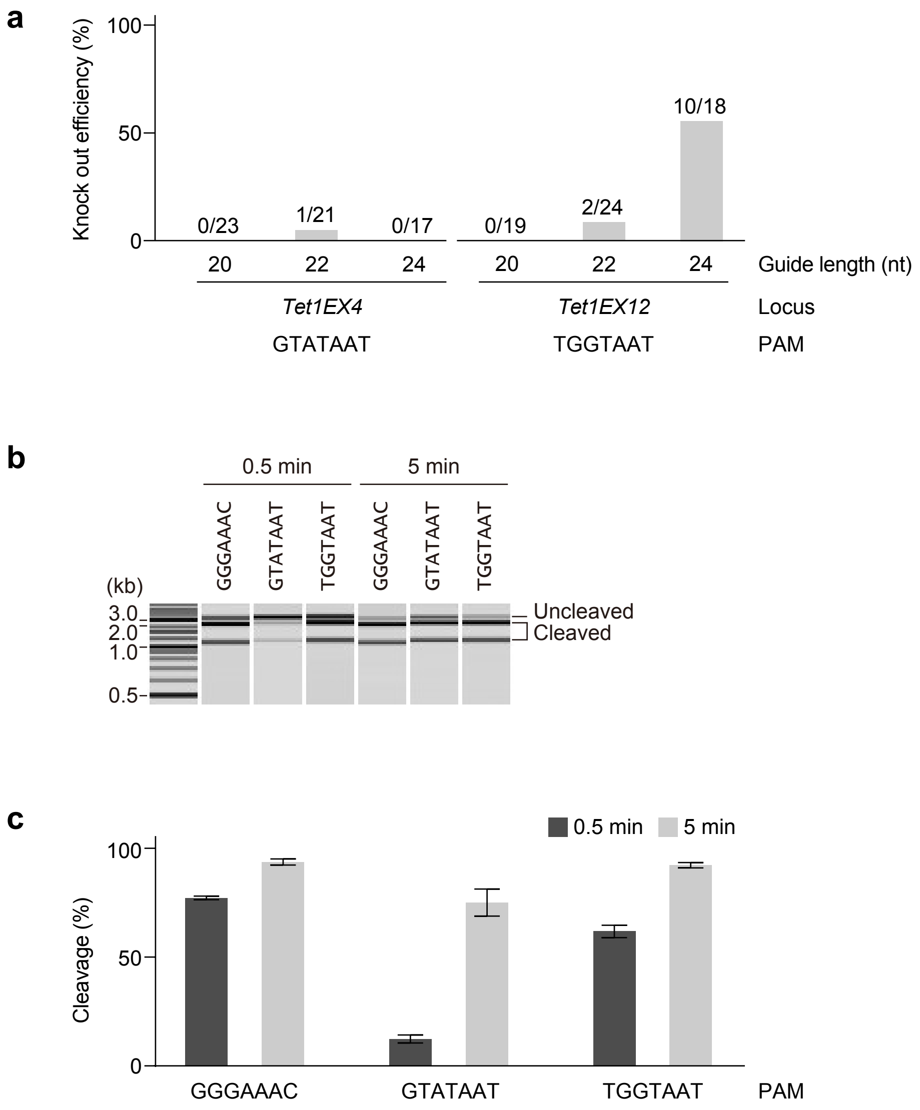

Supplementary Figure 3 | Indel analysis in mouse zygotes.

a Indel analysis in mouse zygotes. The ratios between the numbers of embryos with CdCas9-induced indels and the total numbers of injected embryos are shown above the bars.

b *In vitro* cleavage activities of CdCas9 toward the plasmid targets with the PAMs used in the indel analysis. The linearized plasmid targets bearing the GGGAAAC, GTATAAT, and TGGTAAT PAMs were incubated with the CdCas9-sgRNA22 at 37°C for 0.5 and 5 min. Source data are provided as a Source Data file.

c Quantification of the DNA cleavage activities in (**b**). Error bars represent s.d. from n = 3 replicates.

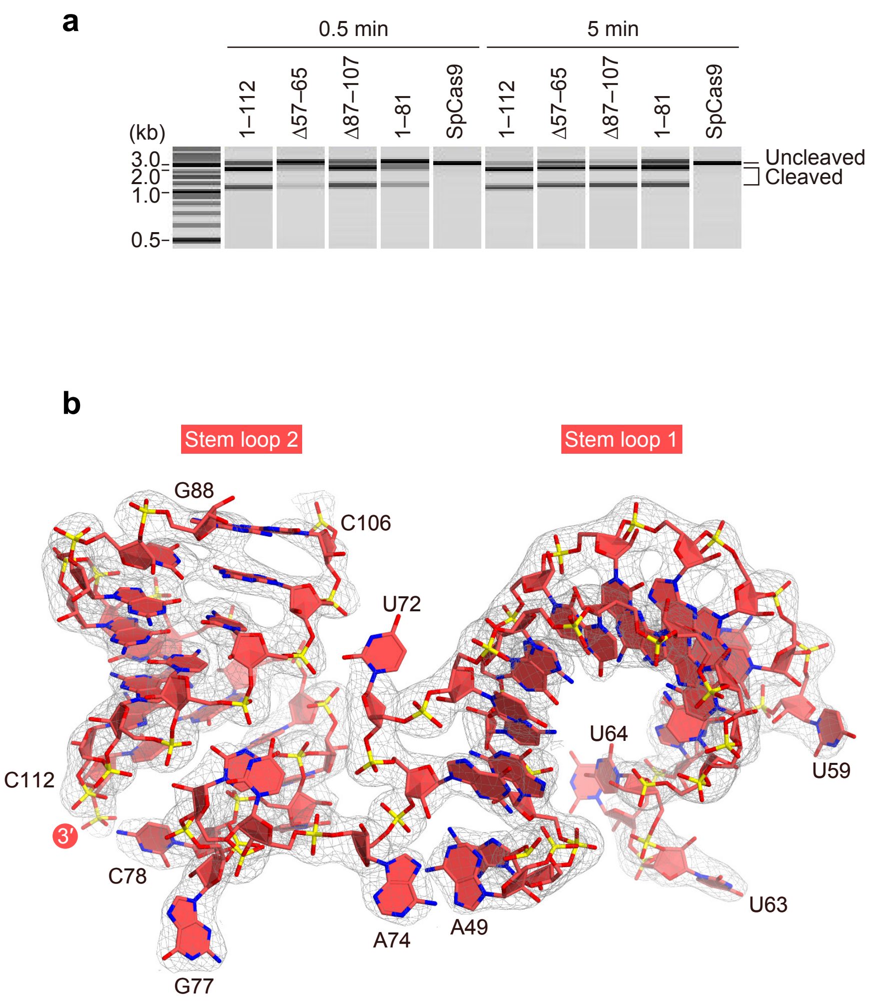

Supplementary Figure 4 | Structure and recognition of the sgRNA scaffold.

a DNA cleavage time courses of CdCas9 with the truncated CdCas9 sgRNAs or the SpCas9 sgRNA, toward the linearized plasmid target with the GGGAAAC PAM (0.5 and 5 min).1–112, the full-length sgRNA; Δ57–65, the sgRNA, in which nucleotides 57–65 were replaced with GAAA; Δ87–107, the sgRNA, in which nucleotides 87–107 were replaced with GAAA; 1–81, the sgRNA, in which nucleotides 82–112 were truncated; SpCas9, the SpCas9 sgRNA.

b $2mF_O - DF_C$ electron density map for the sgRNA scaffold (contoured at 1.5σ). The guide segment and the repeat:anti-repeat duplex are omitted for clarity.

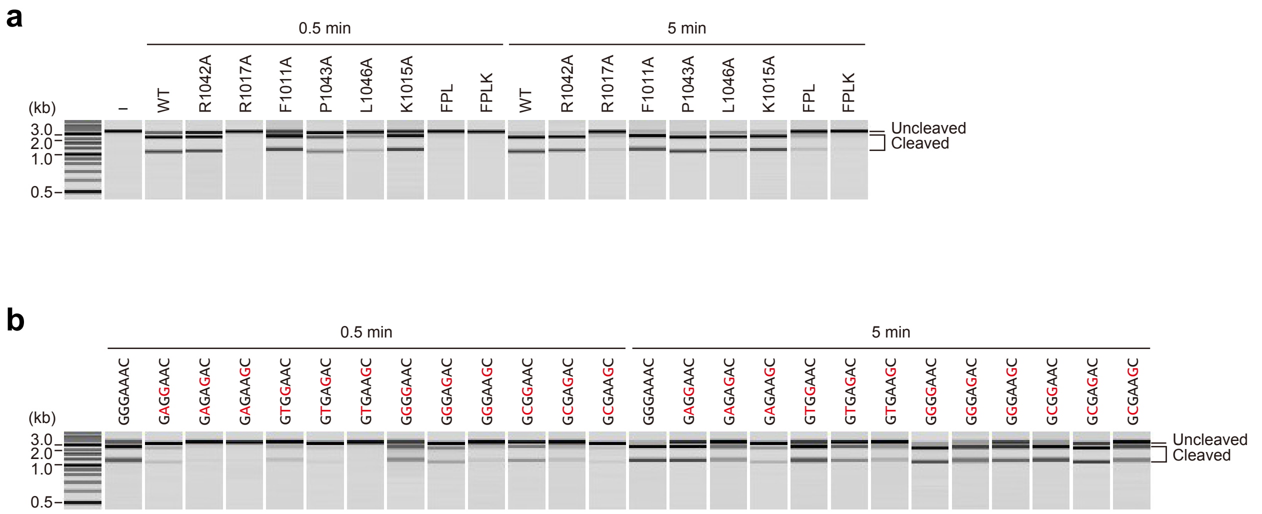

Supplementary Figure 5 | PAM recognition by CdCas9.

a DNA cleavage time courses of the wild type (WT) and the mutants of CdCas9 with the sgRNA22 toward the linearized plasmid target with the GGGAAAC PAM (0.5 and 5 min). FPL, F1011A/P1043A/L1046A; FPLK, F1011A/K1015A/P1043A/L1046A.

b DNA cleavage time courses of CdCas9-sgRNA22 toward the linearized plasmid targets with the different PAMs (0.5 and 5 min).

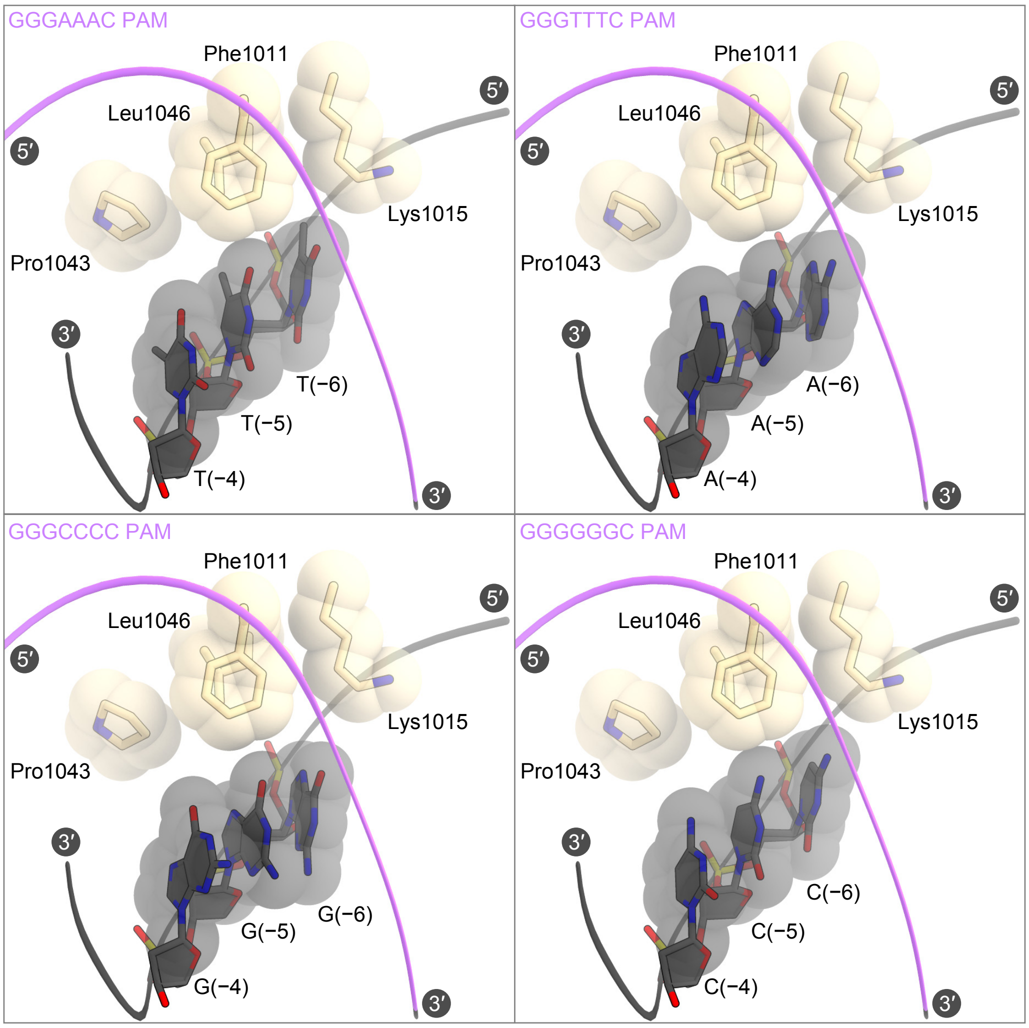

Supplementary Figure 6 | Structural basis for the PAM preference.
Based on the CdCas9 structure with the GGGTAAT PAM, AAA, TTT, CCC, and GGG were modeled at positions 4–6 in the target DNA strand.

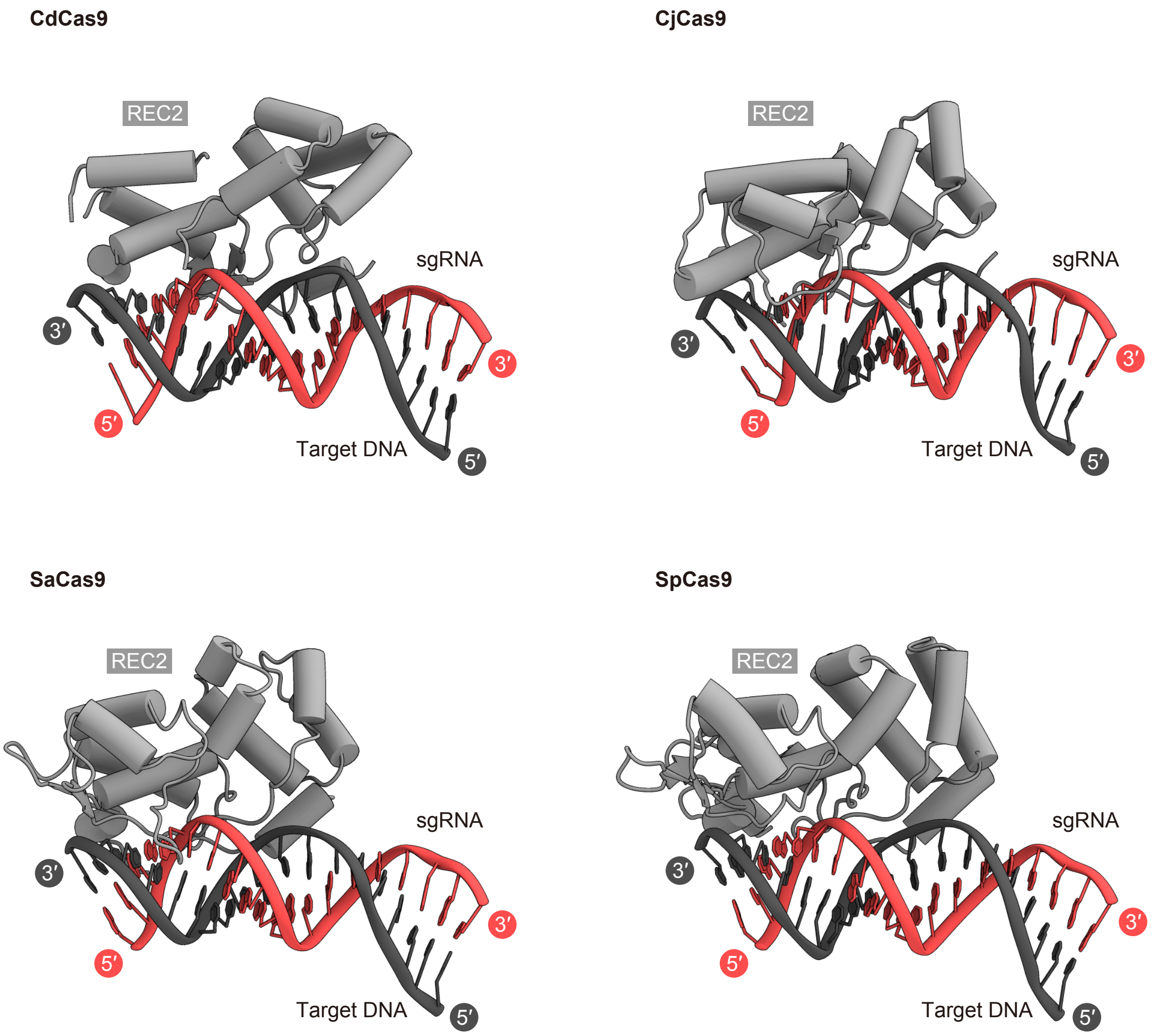

Supplementary Figure 7 | REC2 domains of the Cas9 orthologs.
The REC2 domains of CdCas9, CjCas9 (PDB: 5X2G), SaCas9 (PDB: 5CZZ), and SpCas9 (PDB: 4UN3) with their RNA-DNA heteroduplexes.

Supplementary Table 1 | Target sequences for the indel analysis in human cells

Locus	Target sequence (5′ to 3′)	PAM	Indel (%)
DNMT1	ACCCCTGTTTCTGGCACCAG	GGAATCC	N.D.
DNMT1	TCACCCCTGTTTCTGGCACCAG	GGAATCC	N.D.
DNMT1	CGTCACCCCTGTTTCTGGCACCAG	GGAATCC	N.D.
DNMT1	ATGTTGGGGATTCCTGGTGC	CAGAAAC	N.D.
DNMT1	GCATGTTGGGGATTCCTGGTGC	CAGAAAC	N.D.
DNMT1	GTGCATGTTGGGGATTCCTGGTGC	CAGAAAC	N.D.
DNMT1	GAACGGACAGATTGACATGT	TAAAAAC	N.D.
DNMT1	GTGAACGGACAGATTGACATGT	TAAAAAC	N.D.
DNMT1	ATGTGAACGGACAGATTGACATGT	TAAAAAC	N.D.
DNMT1	ACGGACAGATTGACATGTTA	AAAACAC	N.D.
DNMT1	GAACGGACAGATTGACATGTTA	AAAACAC	N.D.
DNMT1	GTGAACGGACAGATTGACATGTTA	AAAACAC	N.D.
DYRK1A	AAGATTTTCTGAAGATTTTC	TCAATAC	N.D.
DYRK1A	AAAAGATTTTCTGAAGATTTTC	TCAATAC	N.D.
DYRK1A	CAAAAAGATTTTCTGAAGATTTTC	TCAATAC	N.D.
DYRK1A	CTATGGATCTTCTATACCAT	TAAAAAC	N.D.
DYRK1A	CACTATGGATCTTCTATACCAT	TAAAAAC	N.D.
DYRK1A	ATCACTATGGATCTTCTATACCAT	TAAAAAC	N.D.
DYRK1A	GGAACCATTAGATATGTCAA	AAAAAAC	N.D.
DYRK1A	AAGGAACCATTAGATATGTCAA	AAAAAAC	N.D.
DYRK1A	TTAAGGAACCATTAGATATGTCAA	AAAAAAC	N.D.
DYRK1A	ACTTTAGGATATTCAATATT	ATGATAC	N.D.
DYRK1A	GAACTTTAGGATATTCAATATT	ATGATAC	N.D.
DYRK1A	AAGAACTTTAGGATATTCAATATT	ATGATAC	N.D.
EMX1	TCAGCTCAGCCTGAGTGTTG	AGGCCCC	N.D.
EMX1	TCTCAGCTCAGCCTGAGTGTTG	AGGCCCC	N.D.
EMX1	GCTCTCAGCTCAGCCTGAGTGTTG	AGGCCCC	N.D.
EMX1	GGCTGCTCTGGGGGCCTCCT	GAGTTTC	N.D.
EMX1	GTGGCTGCTCTGGGGGCCTCCT	GAGTTTC	N.D.
EMX1	CAGTGGCTGCTCTGGGGGCCTCCT	GAGTTTC	N.D.
EMX1	GCACAGATGAGAAACTCAGG	AGGCCCC	N.D.
EMX1	GGGCACAGATGAGAAACTCAGG	AGGCCCC	N.D.
EMX1	AGGGGCACAGATGAGAAACTCAGG	AGGCCCC	N.D.
EMX1	AGGGAGGGAGGGGCACAGAT	GAGAAAC	N.D.
EMX1	CCAGGGAGGGAGGGGCACAGAT	GAGAAAC	N.D.
EMX1	GGCCAGGGAGGGAGGGGCACAGAT	GAGAAAC	N.D.
DNMT1	ACTTTTCCTTTTATTTCCCTTCAG	CTAAAAT	N.D.
DNMT1	TCCTCCTCCTTTATTTTAGCTGAA	GGGAAAT	N.D.
DYRK1A	TAAAATGAATATAAACTTGTATTG	AGAAAAT	N.D.
DYRK1A	AATGGTATAGAAGATCCATAGTGA	TTAAAAT	N.D.

*N.D., not detected

Supplementary Table 2 | Target sequences for the indel analysis in mouse zygotes

Locus	Target sequence (5′ to 3′)	PAM	Indel (%)	Indel detection
Tet1EX4	TTGGTCCTGCCCCAAGGTGT	GTATAAT	N.D.	*Eco*T14I
Tet1EX4	ACTTGGTCCTGCCCCAAGGTGT	GTATAAT	5	*Eco*T14I
Tet1EX4	ACACTTGGTCCTGCCCCAAGGTGT	GTATAAT	N.D.	*Eco*T14I
Tet1EX12	ACCCTTACCCTGGAGTTCCA	TGGTAAT	N.D.	*Eco*T14I
Tet1EX12	TCACCCTTACCCTGGAGTTCCA	TGGTAAT	8	*Eco*T14I
Tet1EX12	TGTCACCCTTACCCTGGAGTTCCA	TGGTAAT	56	*Eco*T14I
Tet1EX12	TTATAAGTTTGTGTGATAAG	GGGTAAT	N.D.	HMA
Tet1EX12	CGTTATAAGTTTGTGTGATAAG	GGGTAAT	N.D.	HMA
Tet1EX12	TTCGTTATAAGTTTGTGTGATAAG	GGGTAAT	N.D.	HMA
Tet1EX7	CCTGCACCTGTCAAGGCATCGACC	CAAAAAC	N.D.	*Taq*I
Tet1EX12	AGCCTAATCATGGTTTTGATATCA	ACAAAAT	N.D.	*Eco*RV
Tet1EX12	TATCAATTTTCCCTCTTTAACTAG	AAAAAAT	N.D.	*Bfa*I
Tet1EX12	AAAAGTTCCCAGTGTTTCAAGATT	AGAAAAT	N.D.	*Hpy*188III
Tet1EX12	GATTATCATTTCACAAGAACATGT	ACAAAAC	N.D.	*Nla*III

*N.D., not detected

Supplementary Table 3 | Amino acid sequences of the CdCas9 proteins used in this study

Full-length CdCas9:

MKYHVGIDVGTFSVGLAAIEVDDAGMPIKTLSLVSHIHDSGLDPDEIKSAVTRLASSGIARRTRRLYRRKRRRLQQLDKFIQRQGWPVIELEDYSD
PLYPWKVRAELAASYIADEKERGEKLSVALRHIARHRGWRNPYAKVSSLYLPDGPSDAFKAIREEIKRASGQPVPETATVGQMVTLCELGTLKLRG
EGGVLSARLQQSDYAREIQEICRMQEIGQELYRKIIDVVFAAESPKGSASSRVGKDPLQPGKNRALKASDAFQRYRIAALIGNLRVRVDGEKRILS
VEEKNLVFDHLVNLTPKKEPEWVTIAEILGIDRGQLIGTATMTDDGERAGARPPTHDTNRSIVNSRIAPLVDWWKTASALEQHAMVKALSNAEVDD
FDSPEGAKVQAFFADLDDDVHAKLDSLHLPVGRAAYSEDTLVRLTRRMLSDGVDLYTARLQEFGIEPSWTPPTPRIGEPVGNPAVDRVLKTVSRWL
ESATKTWGAPERVIIEHVREGFVTEKRAREMDGDMRRRAARNAKLFQEMQEKLNVQGKPSRADLWRYQSVQRQNCQCAYCGSPITFSNSEMDHIVP
RAGQGSTNTRENLVAVCHRCNQSKGNTPFAIWAKNTSIEGVSVKEAVERTRHWVTDTGMRSTDFKKFTKAVVERFQRATMDEEIDARSMESVAWMA
NELRSRVAQHFASHGTTVRVYRGSLTAEARRASGISGKLKFFDGVGKSRLDRRHHAIDAAVIAFTSDYVAETLAVRSNLKQSQAHRQEAPQWREFT
GKDAEHRAAWRVWCQKMEKLSALLTEDLRDDRVVVMSNVRLRLGNGSAHKETIGKLSKVKLSSQLSVSDIDKASSEALWCALTREPGFDPKEGLPA
NPERHIRVNGTHVYAGDNIGLFPVSAGSIALRGGYAELGSSFHHARVYKITSGKKPAFAMLRVYTIDLLPYRNQDLFSVELKPQTMSMRQAEKKLR
DALATGNAEYLGWLVVDDELVVDTSKIATDQVKAVEAELGTIRRWRVDGFFSPSKLRLRPLQMSKEGIKKESAPELSKIIDRPGWLPAVNKLFSDG
NVTVVRRDSLGRVRLESTAHLPVTWKVQ

CdCas9-ΔHNH:

MKYHVGIDVGTFSVGLAAIEVDDAGMPIKTLSLVSHIHDSGLDPDEIKSAVTRLASSGIARRTRRLYRRKRRRLQQLDKFIQRQGWPVIELEDYSD
PLYPWKVRAELAASYIADEKERGEKLSVALRHIARHRGWRNPYAKVSSLYLPDGPSDAFKAIREEIKRASGQPVPETATVGQMVTLCELGTLKLRG
EGGVLSARLQQSDYAREIQEICRMQEIGQELYRKIIDVVFAAESPKGSASSRVGKDPLQPGKNRALKASDAFQRYRIAALIGNLRVRVDGEKRILS
VEEKNLVFDHLVNLTPKKEPEWVTIAEILGIDRGQLIGTATMTDDGERAGARPPTHDTNRSIVNSRIAPLVDWWKTASALEQHAMVKALSNAEVDD
FDSPEGAKVQAFFADLDDDVHAKLDSLHLPVGRAAYSEDTLVRLTRRMLSDGVDLYTARLQEFGIEPSWTPPTPRIGEPVGNPAVDRVLKTVSRWL
ESATKTWGAPERVIIEHGGGSGGSMESVAWMANELRSRVAQHFASHGTTVRVYRGSLTAEARRASGISGKLKFFDGVGKSRLDRRHHAIDAAVIAF
TSDYVAETLAVRSNLKQSQAHRQEAPQWREFTGKDAEHRAAWRVWCQKMEKLSALLTEDLRDDRVVVMSNVRLRLGNGSAHKETIGKLSKVKLSSQ
LSVSDIDKASSEALWCALTREPGFDPKEGLPANPERHIRVNGTHVYAGDNIGLFPVSAGSIALRGGYAELGSSFHHARVYKITSGKKPAFAMLRVY
TIDLLPYRNQDLFSVELKPQTMSMRQAEKKLRDALATGNAEYLGWLVVDDELVVDTSKIATDQVKAVEAELGTIRRWRVDGFFSPSKLRLRPLQMS
KEGIKKESAPELSKIIDRPGWLPAVNKLFSDGNVTVVRRDSLGRVRLESTAHLPVTWKVQ

*The linker sequence is highlighted in red.

Supplementary Table 4 | DNA sequence of the full-length CdCas9 used in this study

ATGAAGTACCATGTCGGAATCGATGTCGGAACCTTTTCTGTGGGGCTGGCTGCTATTGAAGTGGATGACGCTGGAATGCCTATTAAGACCCTGAGT
CTGGTGTCACACATTCATGACTCAGGACTGGATCCTGACGAGATCAAGAGCGCTGTGACCAGGCTGGCAAGCTCCGGAATCGCCCGGAGAACAAGG
CGCCTGTACCGACGGAAGAGAAGGCGCCTGCAGCAGCTGGATAAGTTCATCCAGAGGCAGGGCTGGCCAGTGATCGAGCTGGAAGATTACAGCGAC
CCCCTGTATCCTTGGAAGGTGCGCGCCGAACTGGCCGCTTCTTATATTGCTGACGAGAAGGAACGGGGGGAGAAACTGAGTGTGGCTCTGAGACAC
ATCGCAAGGCATCGCGGATGGAGGAACCCTTACGCCAAGGTGTCTAGTCTGTATCTGCCAGATGGCCCCTCAGACGCCTTCAAGGCTATTAGGGAG
GAAATCAAACGCGCTAGCGGCCAGCCTGTGCCAGAGACTGCAACCGTCGGGCAGATGGTGACCCTGTGCGAACTGGGCACACTGAAGCTGCGAGGA
GAGGGAGGAGTGCTGAGTGCACGGCTGCAGCAGTCAGATTACGCCCGCGAGATCCAGGAAATTTGTCGAATGCAGGAGATCGGCCAGGAACTGTAT
CGCAAGATCATTGACGTGGTGTTCGCAGCCGAGTCCCCAAAGGGCTCTGCCTCAAGCCGGGTGGGGAAAGATCCTCTGCAGCCAGGAAAGAACAGA
GCACTGAAAGCCAGCGACGCTTTTCAGCGATACCGGATTGCTGCACTGATCGGCAATCTGAGAGTCAGGGTGGATGGGGAGAAGAGGATTCTGAGC
GTGGAGGAGAAGAACCTGGTGTTCGACCACCTGGTGAATCTGACTCCAAAGAAAGAGCCCGAATGGGTGACCATCGCCGAAATTCTGGGCATCGAT
CGCGGGCAGCTGATCGGAACAGCTACTATGACCGACGATGGAGAGCGAGCAGGAGCCCGACCCCCTACACACGATACTAACAGAAGTATTGTGAAC
AGCCGGATCGCACCACTGGTCGACTGGTGGAAAACAGCTAGCGCACTGGAGCAGCACGCCATGGTGAAGGCACTGTCCAACGCCGAAGTCGACGAT
TTTGATTCTCCCGAGGGAGCAAAAGTGCAGGCATTCTTTGCCGATCTGGACGATGACGTCCACGCCAAGCTGGACAGCCTGCATCTGCCTGTGGGA
CGAGCAGCTTACTCCGAGGACACTCTGGTCAGACTGACCCGACGGATGCTGAGTGATGGGGTGGACCTGTATACCGCCCGGCTGCAGGAGTTCGGA
ATTGAACCTAGCTGGACCCCACCCACACCAAGAATCGGAGAGCCTGTCGGCAATCCAGCCGTCGACCGGGTGCTGAAAACAGTGAGCAGATGGCTG
GAATCCGCAACAAAGACTTGGGGCGCCCCAGAGAGGGTCATCATTGAGCACGTGCGCGAAGGCTTCGTCACTGAGAAACGCGCTCGAGAAATGGAT
GGGGACATGAGAAGGCGCGCAGCCCGGAACGCCAAGCTGTTTCAGGAGATGCAGGAAAAGCTGAATGTGCAGGGCAAACCCAGTCGAGCCGATCTG
TGGAGATACCAGTCAGTGCAGAGACAGAACTGCCAGTGTGCCTATTGCGGGTCCCCAATTACCTTTTCTAATAGTGAAATGGACCACATCGTGCCC
AGAGCAGGGCAGGGATCCACCAACACAAGGGAGAATCTGGTCGCCGTGTGCCATCGCTGTAACCAGTCTAAGGGCAATACACCCTTCGCTATTTGG
GCAAAAAACACTTCTATCGAAGGGGTCAGTGTGAAGGAGGCCGTGGAACGGACCAGACATTGGGTCACTGATACCGGCATGAGAAGCACTGACTTC
AAGAAGTTCACCAAGGCTGTGGTCGAGCGGTTTCAGAGAGCAACAATGGATGAGGAAATCGACGCCAGAAGCATGGAATCCGTCGCCTGGATGGCT
AATGAGCTGAGGAGCCGCGTGGCTCAGCACTTCGCATCCCATGGAACCACAGTCAGGGTGTACCGAGGCAGCCTGACAGCAGAGGCTCGACGGGCA
TCTGGGATCAGTGGAAAGCTGAAATTCTTTGATGGCGTGGGGAAGTCCAGGCTGGATAGAAGGCACCATGCTATTGACGCTGCAGTGATCGCATTC
ACCTCTGACTATGTGGCCGAAACACTGGCTGTCCGCTCAAACCTGAAACAGAGCCAGGCCCACCGACAGGAGGCTCCTCAGTGGAGAGAGTTCACC
GGCAAGGATGCAGAGCATCGAGCAGCTTGGAGAGTGTGGTGCCAGAAGATGGAAAAACTGAGCGCCCTGCTGACCGAGGACCTGCGAGATGACCGG
GTGGTCGTGATGTCTAACGTGCGACTGCGGCTGGGAAATGGCAGTGCCCACAAGGAAACCATTGGCAAACTGTCAAAGGTGAAACTGTCCTCTCAG
CTGTCAGTCAGCGATATCGACAAAGCAAGTTCAGAGGCCCTGTGGTGTGCTCTGACCAGAGAGCCCGGATTCGATCCTAAGGAAGGCCTGCCCGCT
AACCCTGAGAGACACATCAGGGTGAATGGGACACATGTCTACGCCGGGGACAATATTGGACTGTTTCCAGTGTCAGCAGGAAGCATCGCACTGAGG
GGAGGATACGCAGAGCTGGGCAGCTCCTTCCACCATGCTCGCGTGTATAAAATTACTTCCGGCAAGAAACCCGCATTTGCCATGCTGAGGGTGTAC
ACCATCGATCTGCTGCCTTATCGCAACCAGGACCTGTTTAGCGTGGAACTGAAGCCACAGACAATGTCCATGAGGCAGGCTGAGAAGAAACTGCGC
GACGCTCTGGCAACTGGGAATGCAGAATATCTGGGATGGCTGGTCGTGGATGACGAGCTGGTCGTGGATACATCTAAGATTGCCACTGACCAGGTC
AAAGCAGTGGAGGCCGAACTGGGGACTATCCGCCGATGGCGGGTGGATGGATTCTTTTCCCCCTCTAAACTGAGACTGAGGCCTCTGCAGATGTCC
AAGGAGGGGATCAAGAAAGAGTCCGCTCCCGAACTGTCTAAAATCATTGACAGACCAGGATGGCTGCCCGCCGTGAACAAGCTGTTCTCTGATGGA
AATGTCACCGTCGTGCGGAGAGACTCTCTGGGACGCGTGCGACTGGAGAGTACAGCCCACCTGCCTGTCACTTGGAAGGTGCAG

Supplementary Table 5 | Oligonucleotides used for the construction of the CdCas9 mutants

D10A_Fw	GCGGTCGGAACCTTTTCTGTGGGGCTGGCTGC
D10A_Rv	GATTCCGACATGGTACTTCATATGGGATCCTTG
ΔHNH_Fw	AGTGGAGGCAGCATGGAATCCGTCGCCTGGATGG
ΔHNH_Rv	GCCACCTCCGTGCTCAATGATGACCCTCTCTGGGG
R1042A_Fw	CTAAAATCATTGACGCGCCAGGATGGCTGCCC
R1042A_Rv	GGGCAGCCATCCTGGCGCGTCAATGATTTTAG
R1017A_Fw	CCCTCTAAACTGGCGCTGAGGCCTCTGC
R1017A_Rv	GCAGAGGCCTCAGCGCCAGTTTAGAGGG
F1011A_Fw	TCCCCCTCTAAACTGAGACTGAGGCCTCTG
F1011A_Rv	CGCGAATCCATCCACCCGCCATCGGCGG
P1043A_Fw	GCAGGATGGCTGCCCGCCGTGAACAAG
P1043A_Rv	TCTGTCAATGATTTTAGACAGTTCGGGAGCGGAC
L1046A_Fw	GCGCCCGCCGTGAACAAGCTGTTCTCTGATG
L1046A_Rv	CCATCCTGGTCTGTCAATGATTTTAGACAGTTCGG
K1015A_Fw	CTGAGACTGAGGCCTCTGCAGATGTCC
K1015A_Rv	CGCAGAGGGGGAAAAGAATCCATCCACCCGC
F1011A_K1015A_Rv	CGCAGAGGGGGACGCGAATCCATCCACCCG
P1043A_L1046A_Fw	CTAAAATCATTGACAGAGCAGGATGGGCGCCCG
P1043A_L1046A_Rv	CGGGCGCCCATCCTGCTCTGTCAATGATTTTAG

Supplementary Table 6 | DNA sequence of the target plasmid for *in vitro* cleavage assays

AGCGCCCAATACGCAAACCGCCTCTCCCCGCGCGTTGGCCGATTCATTAATGCAGCTGGCACGACAGGTTTCCCGACTGGAAAGCGGGCAGTGAGC
GCAACGCAATTAATGTGAGTTAGCTCACTCATTAGGCACCCCAGGCTTTACACTTTATGCTTCCGGCTCGTATGTTGTGTGGAATTGTGAGCGGAT
AACAATTTCACACAGGAAACAGCTATGACCATGATTACGCCAAGCTTGCATGCCTGCAGGTCGACTCTAGAGGATCCCCGGGTACCGAGCTCGAAT
TCACTGGCCGTCGTTTTACAACGTCGTGACTGGGAAAACCCTGGCGTTACCCAACTTAATCGCCTTGCAGCACATCCCCCTTTCGCCAGCTGGCGT
AATAGCGAAGAGGCCCGCACCGATCGCCCTTCCCAACAGTTGCGCAGCCTGAATGGCGAATGGCGCCTGATGCGGTATTTTCTCCTTACGCATCTG
TGCGGTATTTCACACCGCATACGTCAAAGCAACCATAGTACGCGCCCTGTAGCGGCGCATTAAGCGCGGCGGGTGTGGTGGTTACGCGCAGCGTGA
CCGCTACACTTGCCAGCGCCCTAGCGCCCGCTCCTTTCGCTTTCTTCCCTTCCTTTCTCGCCACGTTCGCCGGCTTTCCCCGTCAAGCTCTAAATC
GGGGGCTCCCTTTAGGGTTCCGATTTAGTGCTTTACGGCACCTCGACCCCAAAAAACTTGATTTGGGTGATGGTTCACGTAGTGGGCCATCGCCCT
GATAGACGGTTTTTCGCCCTTTGACGTTGGAGTCCACGTTCTTTAATAGTGGACTCTTGTTCCAAACTGGAACAACACTCAACCCTATCTCGGGCT
ATTCTTTTGATTTATAAGGGATTTTGCCGATTTCGGCCTATTGGTTAAAAAATGAGCTGATTTAACAAAAATTTAACGCGAATTTTAACAAAATAT
TAACGTTTACAATTTTATGGTGCACTCTCAGTACAATCTGCTCTGATGCCGCATAGTTAAGCCAGCCCCGACACCCGCCAACACCCGCTGACGCGC
CCTGACGGGCTTGTCTGCTCCCGGCATCCGCTTACAGACAAGCTGTGACCGTCTCCGGGAGCTGCATGTGTCAGAGGTTTTCACCGTCATCACCGA
AACGCGCGAGACGAAAGGGCCTCGTGATACGCCTATTTTTATAGGTTAATGTCATGATAATAATGGTTTCTTAGACGTCAGGTGGCACTTTTCGGG
GAAATGTGCGGGGGGGGGAAATTAGGTGCGCTTGGCGGGAAACCCGCTCATGAGACAATAACCCTGATAAATGCTTCAATAATATTGAAAAAGGAA
GAGTATGAGTATTCAACATTTCCGTGTCGCCCTTATTCCCTTTTTTGCGGCATTTTGCCTTCCTGTTTTTGCTCACCCAGAAACGCTGGTGAAAGT
AAAAGATGCTGAAGATCAGTTGGGTGCACGAGTGGGTTACATCGAACTGGATCTCAACAGCGGTAAGATCCTTGAGAGTTTTCGCCCCGAAGAACG
TTTTCCAATGATGAGCACTTTTAAAGTTCTGCTATGTGGCGCGGTATTATCCCGTATTGACGCCGGGCAAGAGCAACTCGGTCGCCGCATACACTA
TTCTCAGAATGACTTGGTTGAGTACTCACCAGTCACAGAAAAGCATCTTACGGATGGCATGACAGTAAGAGAATTATGCAGTGCTGCCATAACCAT
GAGTGATAACACTGCGGCCAACTTACTTCTGACAACGATCGGAGGACCGAAGGAGCTAACCGCTTTTTTGCACAACATGGGGGATCATGTAACTCG
CCTTGATCGTTGGGAACCGGAGCTGAATGAAGCCATACCAAACGACGAGCGTGACACCACGATGCCTGTAGCAATGGCAACAACGTTGCGCAAACT
ATTAACTGGCGAACTACTTACTCTAGCTTCCCGGCAACAATTAATAGACTGGATGGAGGCGGATAAAGTTGCAGGACCACTTCTGCGCTCGGCCCT
TCCGGCTGGCTGGTTTATTGCTGATAAATCTGGAGCCGGTGAGCGTGGGTCTCGCGGTATCATTGCAGCACTGGGGCCAGATGGTAAGCCCTCCCG
TATCGTAGTTATCTACACGACGGGGAGTCAGGCAACTATGGATGAACGAAATAGACAGATCGCTGAGATAGGTGCCTCACTGATTAAGCATTGGTA
ACTGTCAGACCAAGTTTACTCATATATACTTTAGATTGATTTAAAACTTCATTTTTAATTTAAAAGGATCTAGGTGAAGATCCTTTTTGATAATCT
CATGACCAAAATCCCTTAACGTGAGTTTTCGTTCCACTGAGCGTCAGACCCCGTAGAAAAGATCAAAGGATCTTCTTGAGATCCTTTTTTTCTGCG
CGTAATCTGCTGCTTGCAAACAAAAAAACCACCGCTACCAGCGGTGGTTTGTTTGCCGGATCAAGAGCTACCAACTCTTTTTCCGAAGGTAACTGG
CTTCAGCAGAGCGCAGATACCAAATACTGTCCTTCTAGTGTAGCCGTAGTTAGGCCACCACTTCAAGAACTCTGTAGCACCGCCTACATACCTCGC
TCTGCTAATCCTGTTACCAGTGGCTGCTGCCAGTGGCGATAAGTCGTGTCTTACCGGGTTGGACTCAAGACGATAGTTACCGGATAAGGCGCAGCG
GTCGGGCTGAACGGGGGGTTCGTGCACACAGCCCAGCTTGGAGCGAACGACCTACACCGAACTGAGATACCTACAGCGTGAGCTATGAGAAAGCGC
CACGCTTCCCGAAGGGAGAAAGGCGGACAGGTATCCGGTAAGCGGCAGGGTCGGAACAGGAGAGCGCACGAGGGAGCTTCCAGGGGGAAACGCCTG
GTATCTTTATAGTCCTGTCGGGTTTCGCCACCTCTGACTTGAGCGTCGATTTTTGTGATGCTCGTCAGGGGGGCGGAGCCTATGGAAAAACGCCAG
CAACGCGGCCTTTTTACGGTTCCTGGCCTTTTGCTGGCCTTTTGCTCACATGTTCTTTCCTGCGTTATCCCCTGATTCTGTGGATAACCGTATTAC
CGCCTTTGAGTGAGCTGATACCGCTCGCCGCAGCCGAACGACCGAGCGCAGCGAGTCAGTGAGCGAGGAAGCGGAAG

*The target and PAM sequences are highlighted in red and blue, respectively.

Supplementary Table 7 | Oligonucleotides used for the indel analysis in mouse zygotes

Locus	Target sequence (5′ to 3′)	Forward	Reverse
Tet1EX4	TTGGTCCTGCCCCAAGGTGT	ACCACTCCAAGCCCTTTTCT	AGAACAAAGCCCCTGTGCTA
Tet1EX4	ACTTGGTCCTGCCCCAAGGTGT	ACCACTCCAAGCCCTTTTCT	AGAACAAAGCCCCTGTGCTA
Tet1EX4	ACACTTGGTCCTGCCCCAAGGTGT	ACCACTCCAAGCCCTTTTCT	AGAACAAAGCCCCTGTGCTA
Tet1EX12	ACCCTTACCCTGGAGTTCCA	AGGCAAATTGGGATCAGTCA	AGCAAGTTTGGGTTCCCATT
Tet1EX12	TCACCCTTACCCTGGAGTTCCA	AGGCAAATTGGGATCAGTCA	AGCAAGTTTGGGTTCCCATT
Tet1EX12	TGTCACCCTTACCCTGGAGTTCCA	AGGCAAATTGGGATCAGTCA	AGCAAGTTTGGGTTCCCATT
Tet1EX12	TTATAAGTTTGTGTGATAAG	TGGCCTTATCATTAAAAACACATC	GGATAGAATCGCCTCTTCCTG
Tet1EX12	CGTTATAAGTTTGTGTGATAAG	TGGCCTTATCATTAAAAACACATC	GGATAGAATCGCCTCTTCCTG
Tet1EX12	TTCGTTATAAGTTTGTGTGATAAG	TGGCCTTATCATTAAAAACACATC	GGATAGAATCGCCTCTTCCTG
Tet1EX7	CCTGCACCTGTCAAGGCATCGACC	CCATCTTCCAGGCGTACCT	AGGCACTGCAACAGGGTTAT
Tet1EX12	AGCCTAATCATGGTTTTGATATCA	CCTTTTCGTGTGTCCCTTGT	TCTCGGGTTAAGGTTGATGC
Tet1EX12	TATCAATTTTCCCTCTTTAACTAG	CCCGTTAACCTCAGCCTGTA	TTTGCCTCCTTTTCCTTTGA
Tet1EX12	AAAAGTTCCCAGTGTTTCAAGATT	AGGCAAATTGGGATCAGTCA	AGCAAGTTTGGGTTCCCATT
Tet1EX12	GATTATCATTTCACAAGAACATGT	CTTCCATTCACCCCGTTAAA	TGCACCCACAATCGATAGAA
